# Light–dark O_2_ dynamics in submerged leaves of C_3_ and C_4_ halophytes under increased dissolved CO_2_: clues for saltmarsh response to climate change

**DOI:** 10.1093/aobpla/plu067

**Published:** 2014-11-07

**Authors:** B. Duarte, D. Santos, H. Silva, J. C. Marques, I. Caçador, N. Sleimi

**Affiliations:** 1 Centre of Oceanography of the Faculty of Sciences, University of Lisbon (CO), Campo Grande, 1749-016 Lisbon, Portugal; 2 MARE—Marine and Environmental Sciences Centre, Faculty of Sciences, University of Lisbon, Campo Grande 1749-016 Lisbon, Portugal; 3 Biology Department & Centre for Environmental and Marine Studies (CESAM), University of Aveiro, Campus de Santiago, 3810-193 Aveiro, Portugal; 4 c/o Department of Zoology, Faculty of Sciences and Technology, Institute of Marine Research—Marine and Environment Research Centre (IMAR-CMA), University of Coimbra, 3000 Coimbra, Portugal; 5 UR: MaNE, Faculté des sciences de Bizerte, Université de Carthage, 7021 Jarzouna, Bizerte, Tunisie

**Keywords:** Halophytes, PSII photochemistry, rising CO_2_, underwater photosynthesis.

## Abstract

Waterlogging and submergence are the major constraints to which wetland plants are subjected, with inevitable impacts on their physiology and productivity. Global warming and climate change, as driving forces of sea level rise, tend to increase such submersion periods and also modify the carbonate chemistry of the water column due to the increased concentration of CO_2_ in the atmosphere. In the present work, the underwater O_2_ fluxes in the leaves of two abundant Mediterranean halophytes were evaluated at different levels of dissolved CO_2_. Photosynthetic enhancement due to increased dissolved CO_2_ was confirmed for both *Halimione portulacoides* and *Spartina maritima*, probably due to high tissue porosity, formation of leaf gas films and reduction of the oxygenase activity of Rubisco. Enhancement of the photosynthetic rates in *H. portulacoides* and *S. maritima* was concomitant with an increase in energy trapping and transfer, mostly due to enhancement of the carboxylation reaction of Rubisco, leading to a reduction of the energy costs for carbon fixation. Transposing these findings to the ecosystem, and assuming increased dissolved CO_2_ concentration scenarios, the halophyte community displays a new ecosystem function, increasing the water column oxygenation and thus reinforcing their role as principal primary producers of the estuarine system.

## Abstract (precis)

Global warming and climate change, as driving forces of sea level rise, tend to increase marsh submersion periods and also modify the carbonate chemistry of the water column due to the increased concentration of CO2 in the atmosphere. Photosynthetic enhancement due to increased dissolved CO2 was confirmed for both C3 and C4 halophytes. Transposing these findings to the ecosystem, and assuming increased dissolved CO2 concentration scenarios, the halophyte community displays a new ecosystem function, increasing the water column oxygenation and thus reinforcing their role as principal primary producers of the estuarine system

## Introduction

Wetlands are among the most productive ecosystems of the planet, retaining about one-half to one-third of the carbon fixed and providing important ecosystem services to the estuarine system, namely nutrient regeneration, primary production and shoreline stabilization as well as a habitat for wildlife (Caçador *et al.* 2009). Estuarine wetlands are known for their high productivity, which has been attributed to the high degree of halophyte coverage and diversity, with specific zonation, resulting from inter-specific relationships between species and competition for specific optimal habitats (Mendelssohn and Morris 2000). Another key factor defining species expansion, growth and productivity is their exposure to abiotic stresses, both environmental (for example, climate driven) and anthropogenic (for example, pollution with heavy metals). If the future of these ecosystems is to be predicted in the face of climate change, it is of great importance to understand plant stress responses and adaptations at the molecular, biochemical, cellular and physiological levels (Yamaguchi-Shinozaki and Shinozaki 2006; Urano *et al.* 2010).

Waterlogging and submergence are two major features to which wetland plants, especially salt marsh halophytes, are subjected with severe impacts on their survival and productivity (Bailey-Serres and Voesenek 2008; Pedersen *et al.* 2010). Due to tidal flooding, shoots and leaves can become completely submerged, restricting gas exchange and light harvesting (Colmer and Voesenek 2009). The frequency, duration and depth of tidal flooding can influence species distribution in salt marshes, this being determined by, for example, their ecophysiological tolerance to flooding (Voesenek *et al.* 2004; Pedersen and Colmer 2006). Not only does the slow diffusion of CO_2_ in the aquatic environment limit its uptake by leaves (Smith and Walker 1980), but also the decreased light availability, due to attenuation down the water column, impairs photosynthesis (Sand-Jensen 1989; Pedersen and Colmer 2006). Several plant species have developed leaf adaptations to these constraints in order to enhance underwater CO_2_ uptake by reducing morphologically the boundary layer and cuticle resistance or by acquiring HCO_3_^−^ directly from the water column (Colmer and Pedersen 2008). In submerged leaves of halophytes, CO_2_ limitation becomes severe when concentrations are near air equilibrium, due to the elevated saturation point of underwater photosynthesis (20–75 times the water–air equilibrium conditions; Pedersen *et al.* 2010). Taking into account the present projections made by the Intergovernmental Panel for Climate Change (IPCC), it is expected that the increased levels of atmospheric CO_2_ will lead to an inevitable increase in the dissolved CO_2_ in water bodies (IPCC 2002), in this way altering the availability of CO_2_ underwater.

*Spartina maritima* (L.) Loisel (Poaceae) and *Halimione portulacoides* (L.) Allen (Amaranthaceae) are two of the most abundant and productive halophytic plants present in the Mediterranean estuaries (Duarte *et al.* 2010; Caçador *et al.* 2013). These species typically colonize the lower marsh mudflats and the sides of the network of channels within the marshes, being subjected to tidal flooding twice per day (Duarte *et al.* 2009, 2014*a*). Nevertheless, these are photosynthetically different species: *H. portulacoides* utilizes C_3_ photosynthesis (Duarte *et al.* 2012, 2014*b*), while *S. maritima* is a C_4_ plant (Duarte *et al.* 2013*a*, *b*; Duarte *et al.* 2014*b*). Previous work has shown that these species present very different dynamics under atmospheric CO_2_ enrichment as well as under submersion (Duarte *et al.* 2014*a*, *b*).

In the present paper, we report the O_2_ dynamics in both light-exposed and dark-incubated leaves of C_3_ *H. portulacoides* and C_4_ *S. maritima* under different dissolved CO_2_ concentrations. This has provided insights not only on the species tolerance to submersion but also how CO_2_ can ameliorate the imposed submersion stress and its consequences in the ecosystem services provided, namely in terms of water column oxygenation.

## Methods

### Plant harvest

Intact turfs of the target species were collected at the end of the growing season (October), 1 day before the experiments started, from the Tagus estuary (Alcochete, 38°45′38.78″N, 8°56′7.37″W). All the turfs of the same species were of similar height to ensure similar ages. The intact turfs and their rhizosediment were transported to the laboratory of Marine Botany of the Centre of Oceanography, in air-exposed trays and placed in a FytoScope Chamber (Photon Systems Instruments, Czech Republic), in a photoperiod (16/8 h light/dark) at 20 °C, until the beginning of the experiments. Sediment was supplemented with one-fourth strength Hoagland solution with the salinity adjusted to 20 ‰ (estuarine salinity) to maintain moisture conditions from the field.

### Underwater net photosynthesis and respiration

The experimental set-up was based on that of Colmer and Pedersen (2008) for similar experiments. Fully expanded leaves (*n* = 3) were excised from the base of the stem. In the case of *S. maritima* leaves, samples were sliced into similar rectangular segments (∼5 cm) by cutting their extremities. For *H. portulacoides*, intact excised leaves were used, as their approximate length was 5 cm. The segments were immediately placed in 50-mL plastic gas-tight bottles with rubber stoppers (three bottles containing three leaf segments each, per treatment). The Hoagland solution (¼ strength) was used as the incubation medium with salinity adjusted to 20 ‰ (estuarine salinity) with sea salt mixture and supplemented with KHCO_3_ in order to attain the desired dissolved CO_2_ levels (0.05, 0.5, 1.0 and 2.0 mM) and the pH adjusted to 6 with KOH, according to field measurements (Duarte *et al.* 2014*c*). These four dissolved CO_2_ concentrations correspond to 5.0, 50.0, 100 and 200 μM of HCO_3_^−^, respectively. Three replicate bottles per treatment were maintained in the light (PAR 400 μmol photons m^−2^ s^−1^ inside the bottle), while the remaining three replicate bottles were darkened. Both groups were maintained at 25 °C. Bottles containing only the incubation medium were placed under the same conditions to confirm that the O_2_ concentrations were maintained constant in the absence of leaves. Underwater net photosynthesis (*P*_N_) and respiration (*R*_N_) were measured using a dissolved oxygen electrode (WTW Oxi 330i/SET) after 2 h. All tubes were gently stirred every 30 min to allow a homogeneous distribution of oxygen on the incubation medium (Colmer and Pedersen 2008), as confirmed by the constant O_2_ values verified in the blank tubes (only Hoagland solution at different levels of dissolved CO_2_ without leaves) during the time course. The lack of headspace prevented the escape of gaseous CO_2_. Due to differences in the morphology and succulence of the leaves of the two species, O_2_ fluxes were expressed on the basis of dry weight. To determine the dry weight (DW) of the leaves, 20 samples per species, from the same intact turfs used in the experiments, were collected and dried at 60 °C until constant weight and re-weighted. This normalization was adopted instead of using chlorophyll since during stress conditions chlorophyll content can be affected and thus affect the normalization.

In order to achieve a projection of the O_2_ production/consumption rates in the Tagus estuarine system, the rates determined in this work were combined with the known biomasses and areas colonized by each of the studied species in the Tagus estuary (Caçador *et al.* 2009; Duarte *et al.* 2010; Caçador *et al.* 2013). The computed values were expressed on a daily basis, considering two high tides per day (one in daytime and another during the night) with a maximum period of plant submersion of 3 h (Serôdio and Catarino 2000; Duarte *et al.* 2014*b*). The calculations were made for the four dissolved CO_2_ scenarios tested in the present work and the O_2_ production/consumption rates obtained experimentally.

### PAM fluorometry

Modulated chlorophyll fluorescence measurements were made in attached leaves in the field with a FluoroPen FP100 PAM (Photon Systems Instruments). For chlorophyll fluorescence measurements leaves from the light incubations were used. All the measurements in the dark-adapted state were made after covering the leaves with an aluminium foil for 30 min. The minimal fluorescence (*F*_0_) in the dark-adapted state was determined using the measuring modulated light, which was sufficiently low (<0.1 μmol m^−2^ s^−1^) not to induce any significant variation in fluorescence. The maximal fluorescence level (*F*_M_) in the dark-adapted state was measured by a 0.8-s saturating pulse at 8000 μmol m^−2^ s^−1^. The maximum photochemical efficiency was assessed as (*F*_M_− *F*_0_)/*F*_M_. The same parameters were also measured in light-adapted leaves, with *F*′_0_ being the minimum fluorescence, *F*′_M_ the maximum fluorescence and the operational photochemical efficiency. Rapid light curves (RLCs) measured in dark-adapted leaves were attained using the pre-programmed LC1 protocol of the FluoroPen, consisting of a sequence of pulses from 0 to 500 μmol m^−2^ s^−1^. During this protocol, the *F*_0_ and *F*_M_ as well as the maximum photochemical efficiency were measured. Each *Φ*PSII measurement was used to calculate the electron transport rate (ETR) through Photosystem II (PSII) using the following equation: ETR = *Φ*PSII × PAR × 0.5, where PAR is the actinic photosynthetically active radiation generated by the FluoroPen and 0.5 assumes that the photons absorbed are equally partitioned between PSII and PSI (Genty *et al.* 1989). Without knowledge of the actual amount of light being absorbed, fluorescence measurements can only be used as an approximation for electron transport (Beer *et al.* 1998*a**,* *b*; Runcie and Durako 2004). Rapid light curves were generated from the calculated ETRs and the irradiances applied during the RLC steps. Each RLC was fitted to a double exponential decay function in order to quantify the characteristic parameters, *α* and ETR_max_ (Platt *et al.* 1980). The initial slope of the RLC (*α*) is a measure of the light-harvesting efficiency of photosynthesis and the asymptote of the curve and the maximum rate of photosynthesis (ETR_max_) is a measure of the capacity of the photosystems to utilize the absorbed light energy (Marshall *et al.* 2000). The onset of light saturation (*E*_k_) was calculated as the ratio between ETR_max_ and *α*. Excitation light of 650 nm (peak wavelength) from an array of three light-emitting diodes was focused on the surface of the leaf to provide a homogeneous illumination. Light intensity reaching the leaf was 3000 μmol m^−2^ s^−1^, which was sufﬁcient to generate maximum ﬂuorescence in all individuals. The ﬂuorescence signal is received by the sensor head during recording and is digitized in the control unit using a fast digital converter. The OJIP transient (or Kaustsy curves) depicts the rate of reduction kinetics of various components of PS II. When a dark-adapted leaf is illuminated with a saturating light intensity of 3500 μmol m^−2^ s^−1^, it exhibits a polyphasic rise in fluorescence (OJIP). Each letter reflects distinct inflection in the induction curve. The level O represents all the open reaction centres (RC) at the onset of illumination with no reduction of QA (fluorescence intensity lasts for 10 ms). The rise of transient from O to J indicates the net photochemical reduction of QA (the stable primary electron acceptor of PS II) to QA^−^ (lasts for 2 ms). The phase from J to I was due to all reduced states of closed RCs such as QA^−^ QB^−^, QA QB^2−^ and QA^−^ QB H2 (lasts for 2–30 ms). The level P (300 ms) coincides with maximum concentration of QA^−^ QB2 with the maximally reduced plastoquinol pool. Phase P also reflects a balance between the light incident at the PS II side and the rate of utilization of chemical (potential) energy and the rate of heat dissipation (Zhu *et al.* 2005). From this analysis several photochemical parameters were attained and are summarized in Table 1.

**Table 1.** Summary of fluorometric analysis parameters and their description. NPQ, non-photochemical quenching.

*PSII efficiency*	
*F′*_0_ and *F*_0_	Basal fluorescence under weak actinic light in light- and dark-adapted leaves
*F′*_M_ and *F*_M_	Maximum fluorescence measured after a saturating pulse in light- and dark-adapted leaves
*F′*_v_ and *F*_v_	Variable fluorescence light (*F′*_M_− *F′*_0_) and dark (*F*_M_− *F*_0_)-adapted leaves
PSII operational and maximum quantum yield	Light- and dark-adapted quantum yield of primary photochemistry, equal to the efficiency by which an absorbed photon trapped by the PSII reaction centre will result in the reduction of *Q*_A_ to *Q*^−^_A_
NPQ	
*RLCs*	
rETR	Relative ETR at each light intensity (rETR = QY × PAR × 0.5)
*α*	Photosynthetic efficiency, obtained from the initial slope of the RLC
*OJIP-derived parameters*	
*ψ*_P0_	Maximum yield of primary photochemistry
*ψ*_E0_	Probability that an absorbed photon will move an electron into the ETC
*ψ*_D0_	Quantum yield of the non-photochemical reactions
*φ*_0_	Probability of a PSII-trapped electron to be transported from *Q*_A_ to *Q*_B_
*P*_G_	Grouping probability is a direct measure of the connectivity between the two PSII units (Strasser and Stirbet 2001)
ABS/CS	Absorbed energy flux (*F*_0_)
TR_0_/CS	Trapped energy flux (ABS/CS × *ψ*_P0_)
ET_0_/CS	Electron transport energy flux (*ψ*_P0_ × *φ*_0_ × ABS/CS)
DI_0_/CS	Dissipated energy flux (ABS/CS − TR_0_/CS)
Diving force for photosynthesis (DF ABS)	DF ABS = DF RC + DF (*ψ*_P0_ + DF*φ*)
Driving force for trapping electronic energy (DF *ψ*_P0_)	DF *ψ*_P0_ =log (*ψ*_P0_/(1 − *ψ*_P0_))
Driving force for electron transport (DF*φ*)	DF *φ* = log (*φ*_0_/(1 − *φ*_0_))
Driving force for energy absorption (DF RC)	DF RC = log (RC/ABS)

### Antioxidant enzymatic activities

All enzyme extraction procedures were performed at 4 °C. Briefly, fresh leaves were homogenized in sodium phosphate buffer (50 mM, pH 7.6) with Na-EDTA (0.1 mM) at a ratio of 8 mL per 500 mg fresh weight. The homogenate was centrifuged at 8923 rpm for 20 min, at 4 °C, and the supernatant was used for the enzymatic assays. Enzymatic activity measurements were performed at room temperature (18 °C).

Catalase (CAT) activity was measured according to the method of Teranishi *et al.* (1974) by monitoring the consumption of H_2_O_2_ and consequent decrease in absorbance at 240 nm. (*ɛ* = 39.4 mM^−1^ cm^−1^). The reaction mixture contained sodium phosphate buffer (50 mM, pH 7.6), Na-EDTA (0.1 mM) and H_2_O_2_ (100 mM). The reaction was started with the addition of the extract. Ascorbate peroxidase was assayed according to Tiryakioglu *et al.* (2006). The reaction mixture contained sodium phosphate buffer (50 mM, pH 7.0), H_2_O_2_ (12 mM) and l-ascorbate (0.25 mM). The reaction was initiated with the addition of 100 µL of the enzyme extract. The activity was recorded as the decrease in absorbance at 290 nm, and the amount of ascorbate oxidized was calculated from the molar extinction coefficient of 2.8 mM^−1^ cm^−1^.

Guaiacol peroxidase was measured by the method of Bergmeyer *et al.* (1974) with a reaction mixture consisting of sodium phosphate buffer (50 mM, pH 7.0), H_2_O_2_ (2 mM) and guaiacol (20 mM). The reaction was initiated with the addition of 100 µL of the enzyme extract. The enzymatic activity was measured by monitoring the increase in absorbance at 470 nm (*ɛ* = 26.6 mM^−1^ cm^−1^).

Superoxide dismutase (SOD) activity was assayed according to Marklund and Marklund (1974) by monitoring the reduction of pyrogallol at 325 nm. The reaction mixture contained sodium phosphate buffer (50 mM, pH 7.6), Na-EDTA (0.1 mM), pyrogallol (3 mM) and Milli-Q water. The reaction was started with the addition of 10 µL of the enzyme extract. Control assays were done in the absence of a substrate in order to evaluate the auto-oxidation of the substrates.

All enzymatic activities were expressed as U μg^−1^ of protein, where U, a unit, is the amount of enzyme that catalyses the conversion of 1 μmol of substrate per second. Proteins were determined according to Bradford (1976), using bovine serum albumin as the standard protein.

### Statistical analysis

In order to compare the results of the different ecophysiological parameters between species at different treatments, the Kruskal–Wallis test was employed. Statistical analyses were carried out using the Statistica Software version 10 (StatSoft).

## Results

### Underwater net photosynthesis and respiration

**Figure 1.**
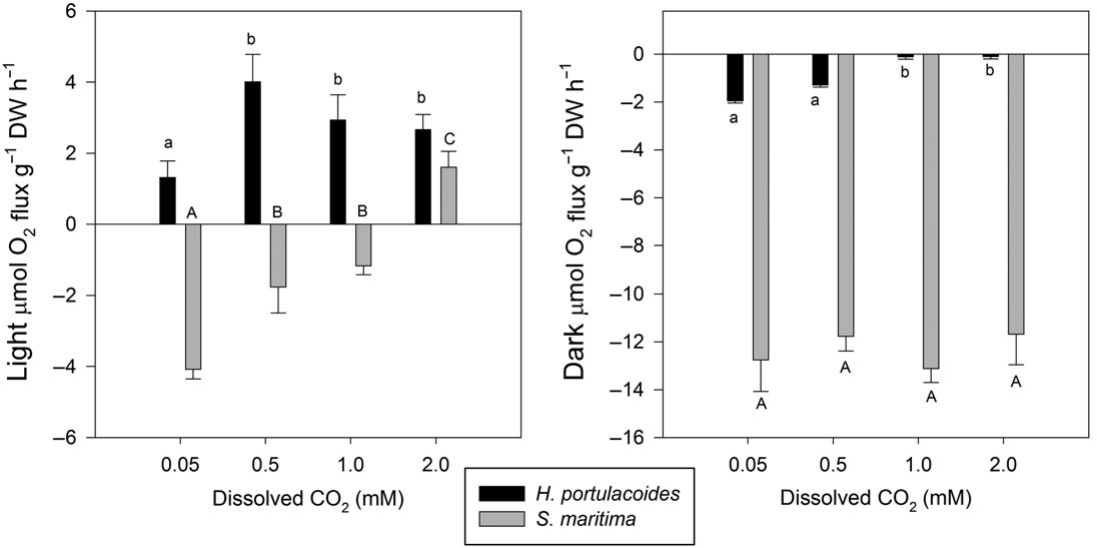
Oxygen production and consumption by the two tested species under light and dark conditions, at different levels of dissolved CO_2_ (average ± standard deviation, *n* = 9. Letters indicate significant differences among CO_2_ treatments at *P*< 0.05).

For *H. portulacoides*, an increase in dissolved CO_2_ led to an increase in O_2_ production (photosynthesis) in the light-exposed leaves and a decrease in the O_2_ consumption (respiration) with increasing dissolved CO_2_ concentrations in dark-incubated leaves (Fig. 1). For *S. maritima*, however, increased dissolved CO_2_ leads to a decrease in O_2_ consumption rates (respiration) in light-exposed leaves. In fact, underwater photosynthetic O_2_ production could only be observed under higher dissolved CO_2_ concentrations. Regarding the dark-adapted leaves of *S. maritima*, there was no distinct pattern of variation in O_2_ consumption rates with change of dissolved CO_2_ concentrations. The highest underwater O_2_ production (4 μmol O_2_ g^−1^ DW h^−1^) was recorded in *H. portulacoides* at 0.5 mM dissolved CO_2_, while *S. maritima* showed the lowest (1.6 μmol O_2_ g^−1^ DW h^−1^) even at elevated dissolved CO_2_ concentrations (2 mM). On the other hand, *S. maritima* exhibited the highest respiratory rates (O_2_ consumption in the dark), independently of the applied dissolved CO_2_ concentration.

### PAM fluorometry

#### PSII quantum efficiencies and variable fluorescence

**Figure 2.**
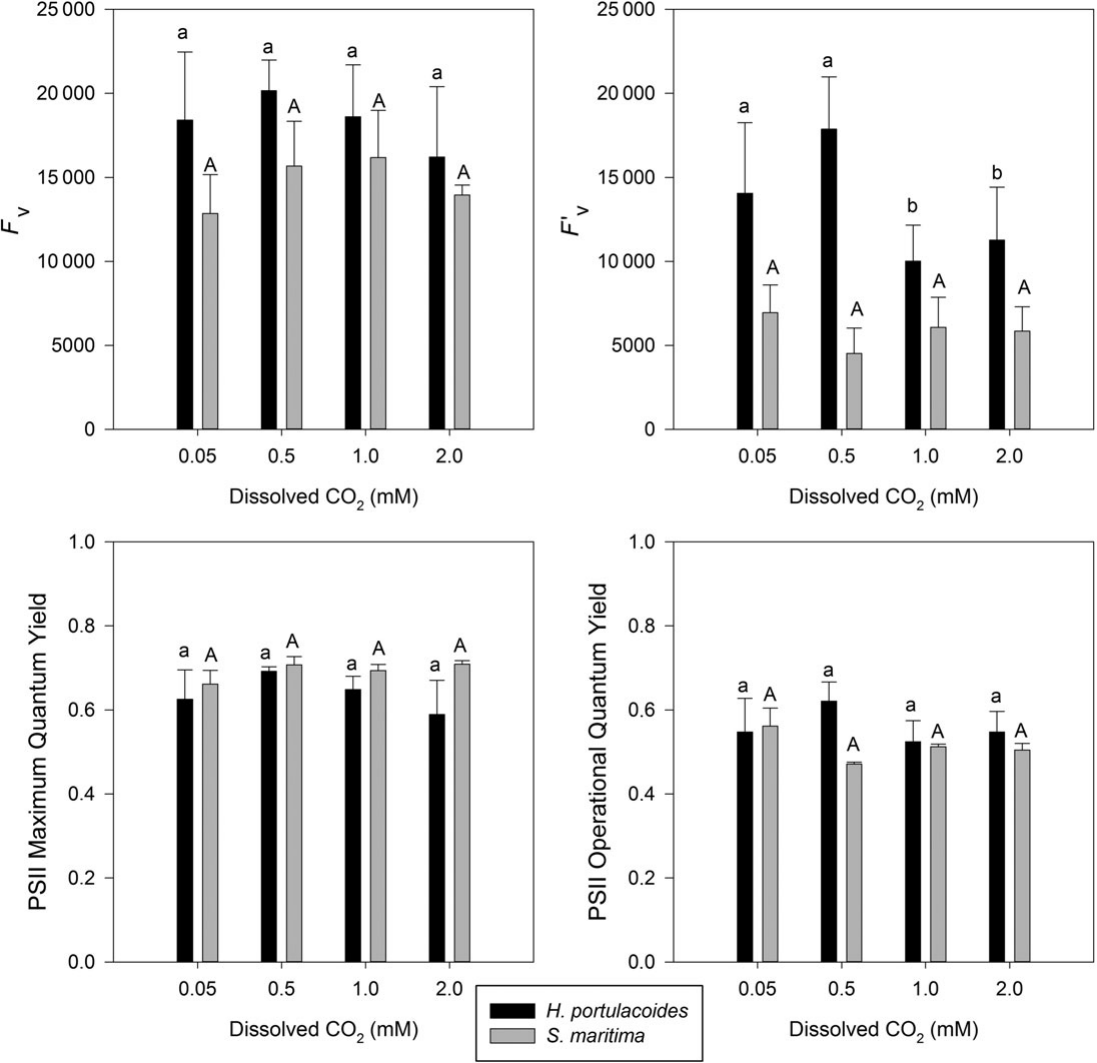
Photosystem II variable fluorescence and quantum efficiencies (operational and maximum) by the two tested species under light and dark conditions, at different levels of dissolved CO_2_ (average ± standard deviation, *n* = 9. Letters indicate significant differences among CO_2_ treatments at *P* < 0.05).

The maximum PSII quantum efficiencies (dark-adapted leaves) showed no evident differences among the dissolved CO_2_ treatments for either species tested (Fig. 2). As for the operational PSII quantum efficiencies, there was a tendency for reduction with increasing dissolved CO_2_ concentrations. The variable fluorescence (*F*_v_) in both dark- and light-adapted leaves of *H. portulacoides* showed a decrease in both with increasing CO_2_ treatments, presenting a maximum at 0.5 mM dissolved CO_2_. On the other hand, in *S. maritima* the *F*_v_ in dark-adapted leaves showed an evident increase along with increasing dissolved CO_2_ concentrations. Nevertheless, there was no distinguishing pattern in the variable fluorescence data in light-adapted leaves of *S. maritima*.

#### Kautsky curves and transient OJIP parameters

**Figure 3.**
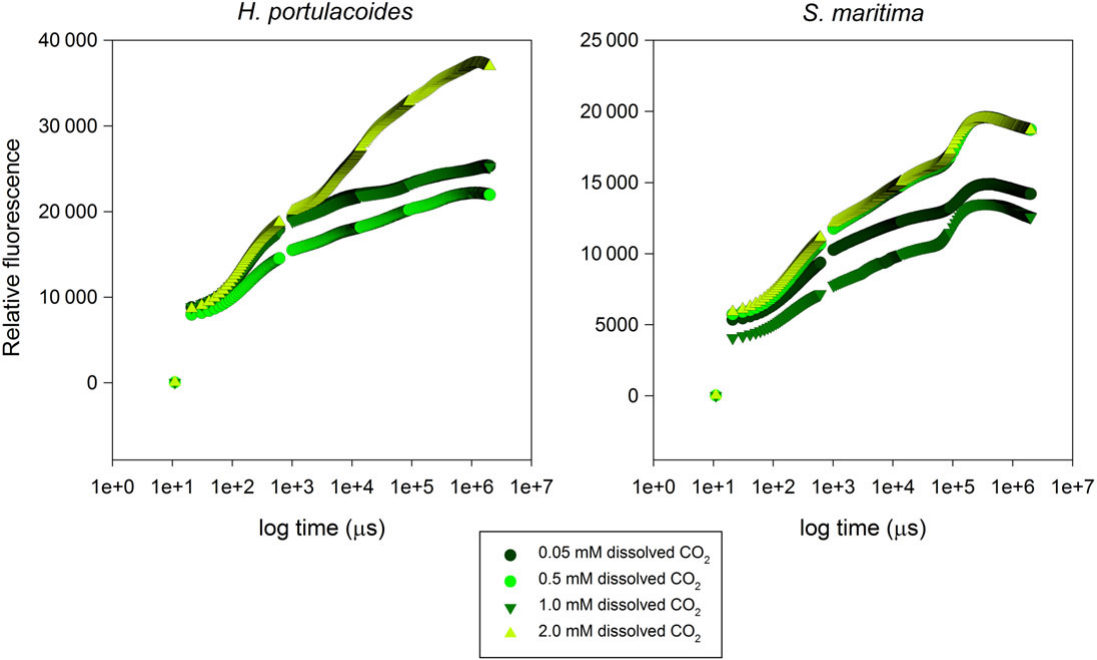
Kautsky OJIP curves of the two tested species under light and dark conditions, at different levels of dissolved CO_2_ (average, *n* = 9).

The O–J phase of the Kautsky curves of all samples presented similar patterns, independently of the dissolved CO_2_ concentrations (Fig. 3). In *H. portulacoides*, the more evident differences were observed during the thermal phase (J–I–P), with higher values in the samples exposed to higher dissolved CO_2_ concentrations. During the photochemical phase (O–J), there was generally an overlap of the Kautsky plots among treatments. For *S. maritima* leaves, the increase in dissolved CO_2_ leads to an increase in the intensity of both the photochemical and thermal phases, the latter being more pronounced in leaves subjected to the higher concentration of dissolved CO_2_.

**Figure 4.**
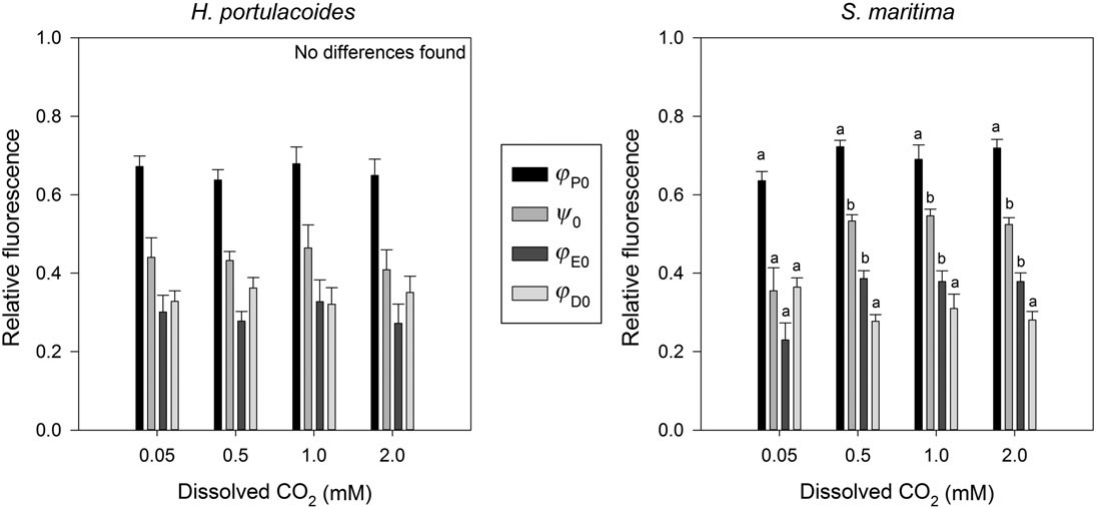
OJIP-driven photochemical parameters in the two tested species under light and dark conditions, at different levels of dissolved CO_2_ (average ± standard deviation, *n* = 9. Letters indicate significant differences among CO_2_ treatments at *P* < 0.05).

Regarding the OJIP-derived photochemical parameters, as well as the energy flux variables, some differences were evident (Figs 3 and 4). The maximum quantum yield of primary PSII photochemistry (*φ*Po) was generally maintained both among CO_2_ treatments and species. On the other hand, in *H. portulacoides*, small fluctuations were observed in the probability of a PSII trapped electron being transferred from QA to QB (*ψ*0) as well as in the electronic transport quantum yield (*φ*Eo) across the different applied CO_2_ treatments. With *S. maritima* both parameters showed an increase in their relative fluorescence with increasing dissolved CO_2_. The quantum yield of the non-photochemical reactions (*φ*Do) decreased only in *S. maritima* with increasing CO_2_.

**Figure 5.**
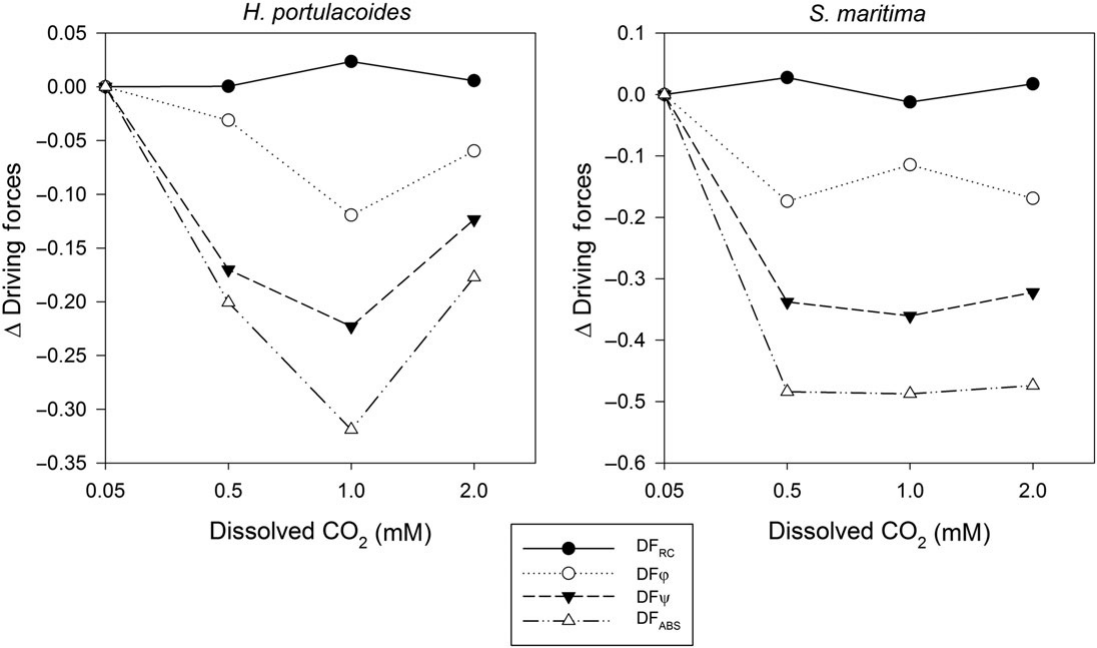
Photochemical reactions driving forces in the two tested species under light and dark conditions, at different levels of dissolved CO_2_ (average, *n* = 9).

If the variations in driving forces at various concentrations of dissolved CO_2_ are analysed (Fig. 5), *H. portulacoides* and *S. maritima* showed a decrease in the driving force for photosynthesis (DF_ABS_) mainly due to a decrease in the trapping (DF*ψ*) of excitation energy and in the conversion of this excitation energy into electron transport (DF*φ*). The DF_RC_ (light energy absorption) was not affected by the increase in dissolved CO_2_, in both these species. Nevertheless in *H. portulacoides*, there was a recovery of the DF_ABS_ at the highest CO_2_ level, driven by a simultaneous increase in both DF*ψ* and DF*φ*.

**Figure 6.**
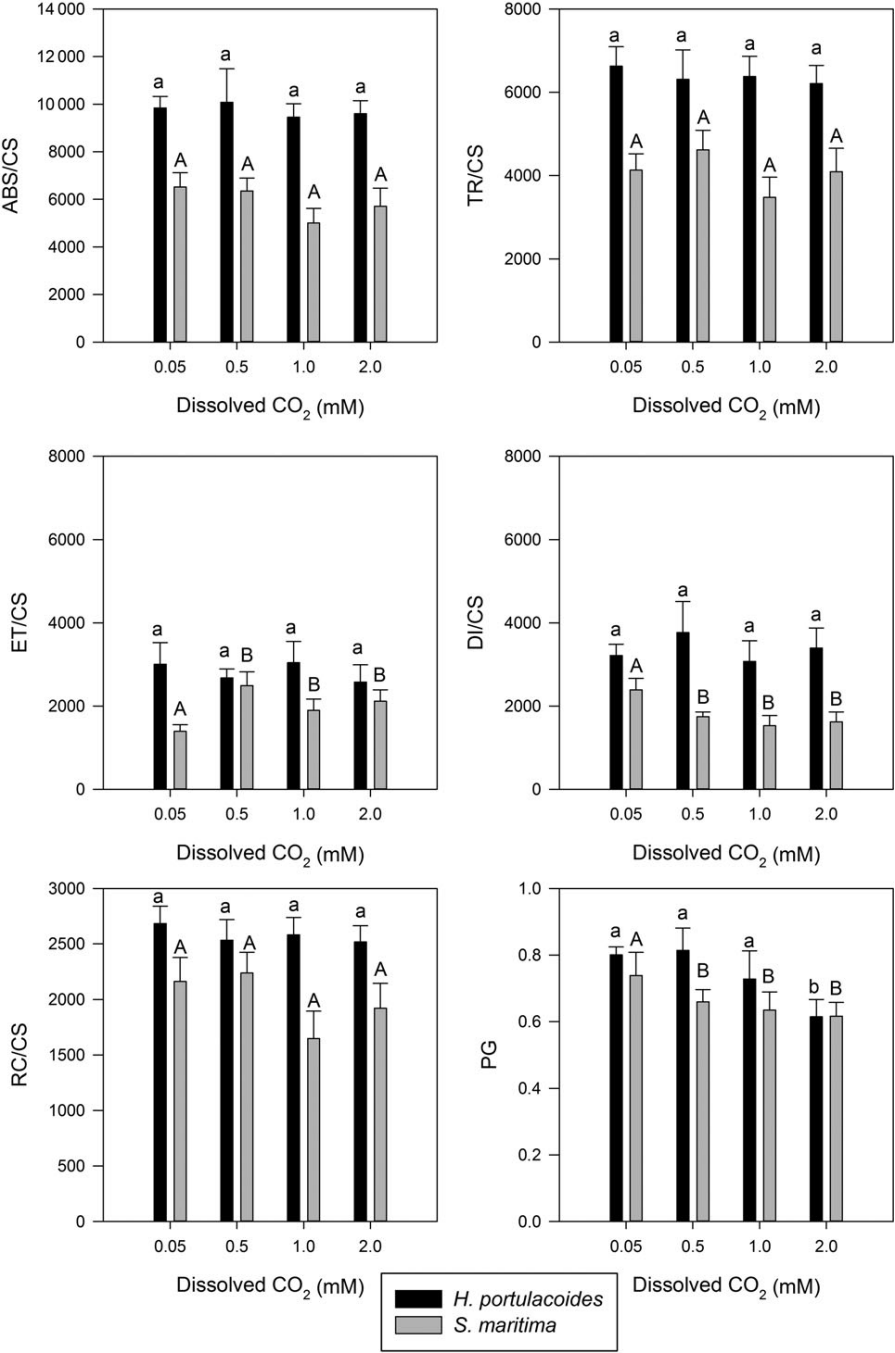
Photosystem II antennae connectivity (PG) and energy fluxes on a leaf cross-section basis, in the leaves of the two tested species under light and dark conditions, at different levels of dissolved CO_2_ (average ± standard deviation, *n* = 9. Letters indicate significant differences among CO_2_ treatments at *P* < 0.05).

Normalizing the data on a leaf cross-section basis provided new insights (Fig. 6). It was possible to see that neither of the species presented differences in the number of available PSII RC for light harvesting (RC/CS), or the absorbed (ABS/CS) or trapped (TR/CS) energy fluxes. On the other hand, the energy fluxes for electron transport (ET/CS) in *S. maritima* showed a marked increase upon CO_2_ supplementation, while *H. portulacoides* leaves did not show any significant difference in this parameter or in the dissipated energy fluxes (DI/CS). *Spartina maritima*, on the other hand, showed a marked reduction in the dissipated energy values at above-ambient CO_2_ concentrations. This analysis also provided insights on the connectivity between the PSII antennae and thus the PSII-harvesting efficiency (PG). While in *H. portulacoides* the increase of the antennae connectivity only occurred at the highest tested CO_2_ concentrations, in *S. maritima* this could be observed in all CO_2_ levels above ambient concentrations.

### Antioxidant enzymatic activities

**Figure 7.**
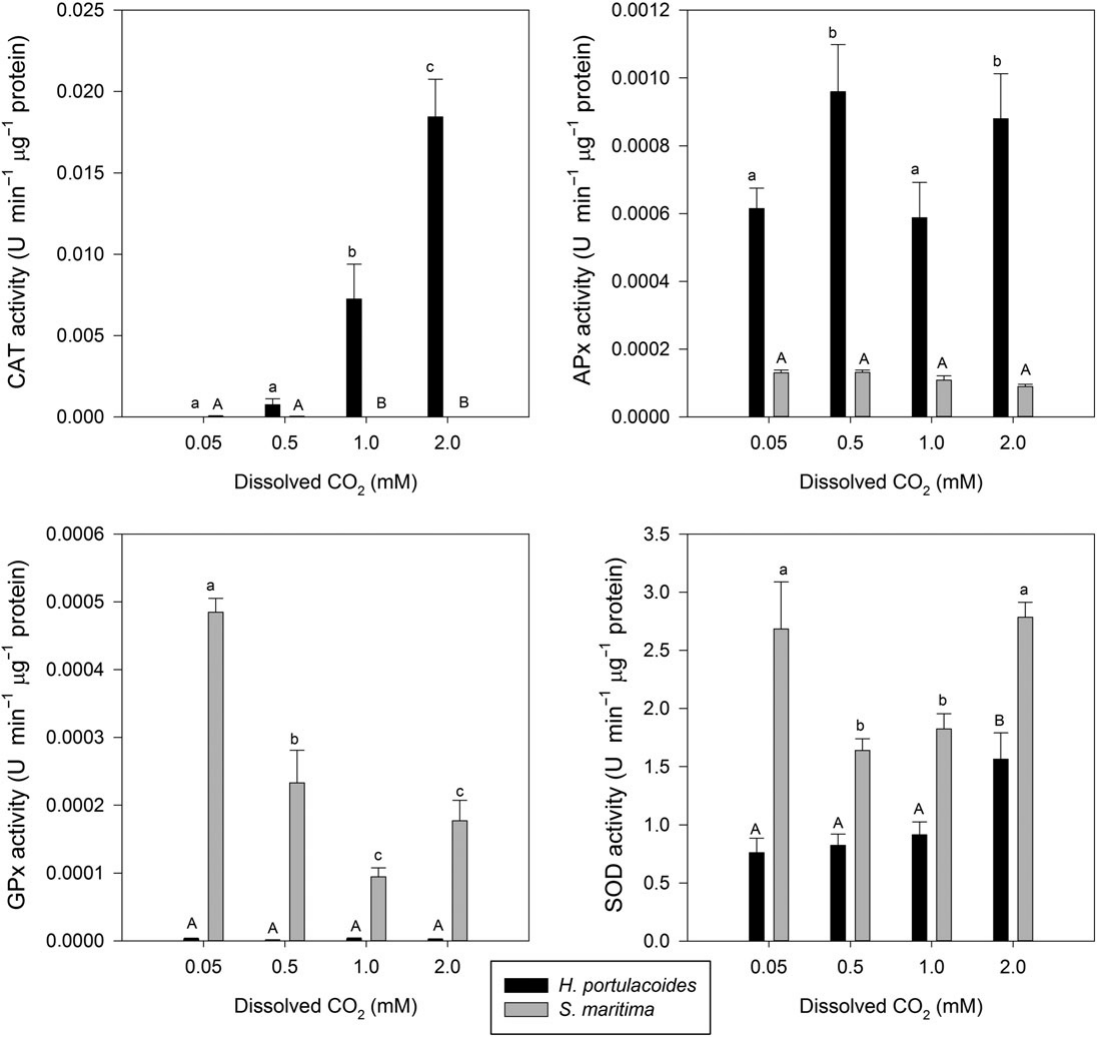
Peroxidase (CAT, APx and GPx) and superoxide dismutase (SOD) activity in the leaves of the two tested species under light and dark conditions, at different levels of dissolved CO_2_ (average ± standard deviation, *n* = 9. Letters indicate significant differences among CO_2_ treatments at *P* < 0.05).

Antioxidant enzymatic defences are presented in Fig. 7. *Halimione portulacoides* showed a marked increase in both CAT and SOD activities with increasing dissolved CO_2_ supply and in *S. maritima* leaves there was a decreasing trend in APx and GPx activities along with increasing dissolved CO_2_. Superoxide dismutase activity increased at the highest dissolved CO_2_ concentration.

### (De)Oxygenation of the water column

Considering the biomass values per square metre and the total abundance of each of the analysed species, the underwater oxygen consumption/production rates were calculated per square metre on a daily basis, for four tested scenarios (Table 2). It was possible to calculate that *S. maritima* was responsible for the highest O_2_ consumptions in the estuarine system. On the other hand, *H. portulacoides* appears as the major contributor for water column oxygenation, with high rates of O_2_ production and very low rates of respiration during nighttime. Overall at the community level, as represented by these two species, an increase in dissolved CO_2_ concentrations in the water column tended to increase the oxygenation of the water column by these halophytes.

**Table 2.** O_2_ (mol) produced (+)/consumed (−) by each halophyte (considering all the coverage area in the Tagus estuary) at the four considered scenarios per day (including light and dark fluxes).

	Dissolved CO_2_ concentration (mM)			
Daytime	0.05	0.5	1	2
* H. portulacoides*	3952 ± 1397	12 050 ± 1397	8816 ± 2140	7990 ± 1301
* S. maritima*	−2039 ± 135	−879 ± 368	−590 ± 124	801 ± 224
Night	0.05	0.5	1	2
* H. portulacoides*	−5872 ± 196	−3882 ± 193	−316 ± 224	−297 ± 210
* S. maritima*	−6379 ± 465	−5885 ± 214	−6557 ± 207	−5840 ± 449
Daily budget	0.05	0.5	1	2
	−10 338	1404	1353	2654

## Discussion

Aquatic plants submerged in different coastal ecosystems are often flooded by water with CO_2_ concentrations above the air–water equilibrium (Sand-Jensen *et al.* 1992; Keeley 1998; Ram *et al.* 1999; Pedersen *et al.* 2010; Duarte *et al.* 2014*b*). The dissolved CO_2_ concentration is a key factor, as its availability controls underwater net photosynthesis, in both terrestrial and aquatic plants (Sand-Jensen *et al.* 1992; Colmer and Pedersen 2008). In estuaries populated with halophytes, not only is their growth controlled by dissolved CO_2_, but also their function as oxygen providers/consumers. The tolerance of these plants to submersion has often been described on the basis of a quiescence response—the lack of shoot elongation conserves carbohydrates during submersion periods (Bailey-Serres and Voesenek 2008). At night-time an increased O_2_ deficit arises as another stressor in submerged conditions, due to the plants' dependency on dissolved O_2_ entry from floodwaters for night respiration (Waters *et al.* 1989; Pedersen and Colmer 2006; Pedersen *et al.* 2009).

In the present study, two of the more abundant halophytic species in the Portuguese estuarine ecosystems showed very different feedbacks to increases in dissolved CO_2_. The photosynthetic enhancement due to increased dissolved CO_2_, verified for *H. portulacoides* and *S. maritima*, has already been described for rice and for *Hordeum marinum* (Setter *et al.* 1989; Pedersen *et al.* 2010). These authors suggested that enhancement was driven by the presence of gas films on the leaf surface and high tissue porosity, allowing higher rates of gas exchange according to CO_2_ availability (Pedersen *et al.* 2010). Also, an increase in [CO_2_]/[O_2_] ratio can improve CO_2_-use efficiency, due to a reduction in Rubisco oxygenase activity (Lorimer 1981; Bowes 1993; Andersen and Pedersen 2002). Although this enhancement was detected even at low dissolved CO_2_ concentrations in *H. portulacoides*, for *S. maritima* it was only achieved at high CO_2_ concentrations. *Spartina maritima* proved to be highly adapted to submersion, in line with a recent study that also demonstrated the plasticity of *S. maritima* as an efficient adaptation to flooding, allowing plants to recover quickly from the stress imposed by this condition (Duarte *et al.* 2014*b*). Being a C_4_ species, these results for *S. maritima* point to a reduction in the oxygenase activity of Rubisco as a consequence of the extremely high internal [CO_2_]/[O_2_]; this, together with a high resistance to CO_2_ entry, restricts underwater photosynthesis (Lorimer 1981; Bowes 1993; Andersen and Pedersen 2002). Although *S. maritima* has a high specific leaf area compared with *H. portulacoides*, the presence of a thicker cuticle is probably the factor responsible for this reduced diffusion underwater (Mommer *et al.* 2007). In *S. maritima*, the CO_2_ concentration mechanism occurs in the mesophyll cells catalysed by PEPc supplying the bundle sheath with C4 organic acids, independently of the O_2_ concentration. In sum, C_4_ plants with their Kranz anatomy rely on the preferential assimilation of HCO_3_^−^ (Raven 1970, 1972). Additionally, it should be noted that, under submersion in turbid water, mitochondrial respiration must be included as a possible contributor for O_2_ budgets. Nevertheless, under these conditions, *S. maritima* HCO_3_^−^-pump allowed an enhancement of the photosynthetic O_2_ production at HCO_3_^−^ concentrations as low as 200 μM. Thus, the CO_2_ concentration mechanism in *S. maritima* is apparently highly adapted to underwater conditions, as shown previously (Duarte *et al.* 2014*b*), needing comparatively low HCO_3_^−^ concentrations to efficiently supply the bundle sheath cells with the required CO_2_ concentration for restarting the Calvin cycle. On the other hand, C_3_ plants depend on the availability of CO_2_, and therefore on the leaf gas film formation (Raven 1970, 1972). The presence of wax-rich cuticles has been reported in hydrophobic leaves of *H. portulacoides* (Grossi and Raphel 2003) and can result in an improvement in gas film formation at the leaf surface, thus promoting gas exchange (Setter *et al.* 1989; Wagner *et al.* 2003; Colmer and Pedersen 2008). Nevertheless, the C_3_ mechanism of *H. portulacoides*, dependent on the CO_2_ acquisition, only showed a significant enhancement at 0.5 mM CO_2_ concentration, making it less efficient than the C_4_ of *S. maritima*. All these facts indicate that, while underwater, these species are C_i_-limited at normal inorganic carbon concentration in estuarine water.

Alongside the anatomical differences, the photosynthetic and electronic processes showed marked differences in light-harvesting and processing mechanisms. One important fluorescence parameter, normally associated with stress conditions is the variable fluorescence (*F*_v_). The maximum photosynthetic O_2_ production in *H. portulacoides*, verified at 0.5 mM dissolved CO_2_, also showed the highest value of *F*′_v_, an indicator of low stress conditions (Duarte *et al.* 2012). The same was true, but to a smaller extent, for the operational quantum yield of this species.

Since CO_2_ and O_2_ compete for the same Rubisco active sites (Taiz and Zeiger 2002), favouring the carboxylation process and reduction of the oxygenation capacity of Rubisco also implies a decrease in the energy costs for CO_2_ fixation and a consequent increase in PSII quantum yield for photosynthesis (Furbank 1998; Taiz and Zeiger 2002). This was confirmed by analysing the principal driving forces underlying the electronic photosynthetic processes. In *H. portulacoides* and *S. maritima* there was not only an increase in the driving forces related to the trapping of excitation energy, but also in transfer of this energy to the electron transport chain. Both these increases lead to an enhancement in total driving force for photosynthesis. It was also interesting to note that, in both species, there were no changes in the light energy absorption ability, confirming the above-mentioned hypothesis (increased PSII efficiency induced by higher Rubisco carboxylation rates) and pointing to an increase in photosynthetic activity due to higher CO_2_ availability. In fact, in *S. maritima* this was related to an increase in the probability of a PSII trapped electron being transferred from QA to QB (*ψ*0) as well as for the quantum yield of this transport between quinones (*φ*Eo).

All these findings are confirmed by the analysis of the energy fluxes on a cross-sectional area basis. Both species maintained their number of available PSII RC constants and thus the absorbed and trapped energy fluxes did not suffer any changes. Differences arise if the integrity of the PSII antennae is observed and are the basis of the increased energy processing efficiency observed in *S. maritima* leaves. This parameter accounts for all the energetic communication pathways between neighbour PSII antennae (Strasser and Stirbet 2001; Panda *et al.* 2006). On the contrary to what has been observed in other terrestrial plants, there was no loss of connectivity between the antennae of the PSII units during submersion, indicating an improved survival strategy for underwater conditions (Panda *et al.* 2006; Duarte *et al.* 2014*b*). The increased PSII antennae integrity in *S. maritima* leads to an enhanced efficiency in transporting energy and thus reduction of the dissipated energy. Although this was also noticed in *H. portulacoides* leaves subjected to the highest dissolved CO_2_ concentrations tested, it did not result in a significant improvement of the energy fluxes use efficiency, indicating that the increasing dissolved CO_2_ concentrations do not affect *H. portulacoides* leaves at the energy processing but in deeper processes.

Comparing the data on antioxidant enzymatic activities with the data from the oxygen production, *H. portulacoides* showed a maximum photosynthetic O_2_ production at 0.5 mM CO_2_ decreasing towards higher CO_2_ concentrations, concomitantly with an increase in CAT activity. This O_2_ production decreased or increased consumption during daytime conditions as well as increased CAT activity, which is in agreement with photorespiratory activation. It is known that activation of photorespiration is an important part of the abiotic stress feedback as a means to dissipate the excessive energy trapped (Voss *et al.* 2013). With the activation of this mechanism, H_2_O_2_ is generated and scavenged by the increased CAT activity. In fact, looking at the data from the principal driving forces, the increase in CAT activity appears simultaneously with a decrease in the driving forces for trapping excitation energy and for photosynthesis. In the C_4_ *S. maritima*, peroxidase activity (APx and GPx) showed a concomitant decrease along with the increase in O_2_ production. On the other hand, SOD showed two marked peaks driven by distinct and opposite mechanisms. The first peak at 0.05 mM CO_2_ is concomitant with high respiratory activity and increased non-photochemical activity, indicating a need to dissipate excessive photon energy, due to the Ci limitation, similar to that observed in plants exposed to increased atmospheric CO_2_ (Geissler *et al.* 2009; Duarte *et al.* 2014*a*). The second observed SOD peak, observed in individuals exposed to 2 mM dissolved CO_2_, is coincident with the increase in the photosynthetic activity, and consequent superoxide radical production (Taiz and Zeiger 2002).

Nevertheless, previous studies (Duarte *et al.* 2014*a*) with CO_2_-enriched air showed a very different trend, pointing to different stress mechanisms, occurring during submersion. While the C_3_ *H. portulacoides* showed an enhancement of the photosynthetic rates while exposed to atmospheric CO_2_ enrichment, the same was not observed for the C_4_ *S. maritima*. In the present study, this grass showed a very significant improvement in its photosynthetic rates upon dissolved CO_2_ fertilization that was not observed in immersed conditions (Duarte *et al.* 2014*b*). This points to two interesting aspects: (i) *S. maritima* is probably *C_i_* limited under submersed conditions and (ii) CO_2_ in fact ameliorates the stress imposed by submersion (Duarte *et al.* 2014*a*).

All these ecophysiological responses have their repercussions for the ecosystem services provided by the salt marshes, namely in terms of O_2_ and CO_2_ production for the water column. Considering the average temperature used in these experiments and the biomass production drawn from the literature (Caçador *et al.* 2009; Duarte *et al.* 2010) as well as the species coverage (Caçador *et al.* 2013), a very simple and basic extrapolation was made for the Tagus estuary salt marshes. It was possible to calculate that there is a general trend for increasing water column oxygenation during the daily tidal cycle (two tides, one in the daytime and another during the night-time), driven by plant underwater photosynthesis. This becomes of great importance if we consider salt marshes as one of the most important primary producers in an estuarine system (Caçador *et al.* 2007, 2013; Duarte *et al.* 2010). Oxygen production by the halophytes becomes a key player overcoming the low rates of air–water O_2_ diffusion, and thus introduces important amounts of oxygen required for the heterotrophic species (fishes, macro-invertebrates and bacteria). According to this conclusion, a new saltmarsh service arises as a crucial O_2_ producer for the estuarine aquatic community to accompany the role of these marshes as important carbon-harvesting primary producers.

## Conclusions

The diurnal tidal flooding imposes on the halophyte community an underwater environment where gas concentrations are very different from the atmospheric ones. The predicted atmospheric CO_2_ increase will have consequences not only on the atmospheric composition, but also on the carbonate chemistry of estuarine water. It is important to consider the physiology of each species, and the consequences that their adaptations to altered CO_2_ concentration have in terms of the ecosystem they inhabit. The presence of leaf gas films, lack of tissue porosity or other morphological traits of the leaf, as well as photochemical differences and biochemical responses to the imposed condition, are very important characteristics that from a holistic point of view have enormous impacts on the estuarine water column chemistry as a habitat for heterotrophic species. Salt marshes will play a crucial role in counterbalancing the effects of climate change, in terms of water column oxygenation and in buffering its acidification by withdrawing excess CO_2_.

## Sources of Funding

The work was funded by ‘Fundação para a Ciência e Tecnologia (FCT)’ at the Centre of Oceanography (CO) throughout the project PEst-OE/MAR/UI0199/2011, the Institute of Marine Research (IMAR) throughout the project PEst-C/MAR/UI0284/2011 and this specific work throughout the ECOSAM project (PTDC/AAC-CLI/104085/2008). The investigation of B.D. was supported by FCT through a PhD grant (SFRH/BD/75951/2011).

## Contributions by the Authors

In the present work, all the authors were involved according to their area of expertise. B.D. was responsible for the biophysical and photochemical analyses and D.S. for the antioxidant enzymatic assays. I.C. and J.C.M. were responsible for the supervision of the work and for the final corrections and suggestions in data interpretation. H.S. and N.S. supervised the work and introduced their expertise in terms of halophyte ecology.

## Conflicts of Interest Statement

None declared.
